# This Little PBDE Went to Market: Estimating Intake from Grocery Store Foods

**Published:** 2006-10

**Authors:** Victoria McGovern

High concentrations of polybrominated diphenyl ethers (PBDEs) found in the U.S. population are a cause for concern because of these compounds’ similarity to polychlorinated biphenyls. Unlike the latter, which have recently decreased in blood levels, PBDEs have increased substantially in the last two to three decades. A new U.S. “market basket” survey measuring values of PBDEs in grocery store foods shows which chemicals within this class are taken in by eating and adds to a growing body of evidence that food is only part of how humans are exposed to these chemicals **[*EHP* 114:1515–1520; Schecter et al.]**. This article is also the first to estimate U.S. PBDE intake via food from infancy to old age.

PBDEs are flame retardants applied to fabrics, incorporated into plastics and electronics, and mixed into the foam cushioning used in furniture. The behavior of PBDE congeners can differ due to variable physical, chemical, and biological properties. Though human health effects are not yet well understood, PBDEs’ reach in animal studies includes reproductive and developmental toxicity, endocrine disruption, cancer, and central nervous system effects. High levels of PBDEs have been found in human milk, blood, and adipose tissue, as well as in food. U.S. blood and breast milk samples have shown levels 10 to 20 times higher than similar samples from Europeans.

The team used high-resolution mass spectrometry to measure 13 different PBDE congeners in samples of 62 basic foods including fresh and processed meats, fish, milk products, and eggs. The foods analyzed were purchased at three large national chain supermarkets in Dallas in 2003 and 2004.

Of the 13 congeners measured, only about half were found as major contaminants of the food sampled, a finding that parallels earlier observations of the relative prevalence of various congeners in human blood. Although levels of PBDEs varied greatly even within samples of the same type of food, some trends were clear: fish had the most PBDE contamination by weight, followed by meats and dairy foods. But when relative consumption of these foods by Americans was taken into account, meat contributed the most PBDEs to the diet of Americans beyond weaning. (Nursing infants’ intake of PBDEs is primarily via breast milk.)

The analysis showed that U.S. foods are generally more contaminated by PBDEs than foods in Japan or Spain, as reported in earlier surveys. But these differences still are not enough to explain the much larger blood and milk burdens observed in Americans. The authors suggest, as others have earlier, that additional routes of exposure, such as house dust inhalation and ingestion, also play important roles in PBDE exposure among Americans.

## Figures and Tables

**Figure:**
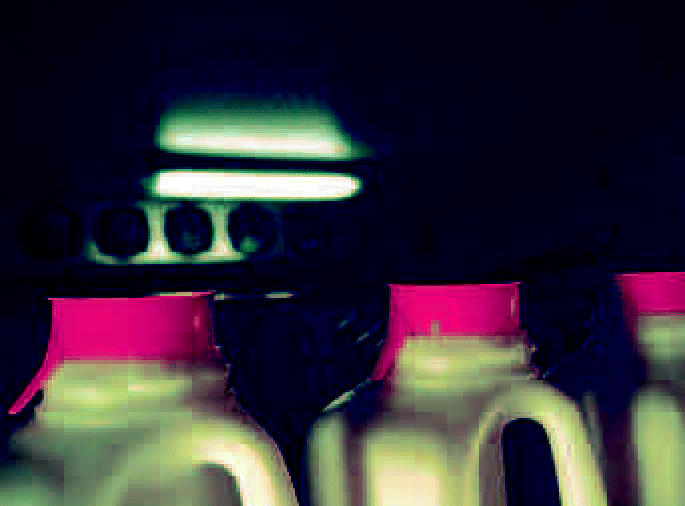
Chemicals in the case New findings show how PBDEs in dairy products, meats, and other foods contribute to levels in Americans’ blood.

